# The Impact of SYNTAX Score of Non-Infarct-Related Artery on Long- Term Outcome among Patients with Acute ST Segment Elevation Myocardial Infarction Undergoing Primary Percutaneous Coronary Intervention

**DOI:** 10.1371/journal.pone.0109828

**Published:** 2014-10-10

**Authors:** Min-I Su, Cheng-Ting Tsai, Hung-I Yeh, Chun-Yen Chen

**Affiliations:** 1 Division of Cardiology, Department of Internal Medicine, Mackay Memorial Hospital, Taipei, Taiwan; 2 Mackay Medical College, New Taipei City, Taiwan; Azienda Ospedaliero-Universitaria Careggi, Italy

## Abstract

**Objective:**

We investigated the impact of the severity of stenosis in a non-infarct-related artery (IRA) on the long-term prognosis of patients with ST-segment elevation myocardial infarction (STEMI) undergoing primary percutaneous coronary intervention (PCI).

**Methods:**

Three hundred one consecutive patients (age: 59.7 ± 13.2 years, 85.5% men) underwent primary PCI during 2009–2012. Receiver operating characteristic curve analysis found the optimal cutoff for non-IRA SYNTAX score (SS) to be 2.5. We divided the patients into two groups according to this cutoff value.

**Results:**

By multivariable analysis, non-IRA SS (≥2.5) was an independent predictor of major adverse cardiac events (hazard ratio [HR]: 2.15, 95% confidence interval [CI]: 1.21–3.79, P  =  0.008) and all-cause mortality (HR: 3.49, 95% CI: 1.13–10.8, P  =  0.03). However, the prediction of cardiovascular mortality had only borderline significance (HR: 3.29, 95% CI: 0.90–12.08, P  =  0.07).

**Conclusion:**

STEMI patients treated with primary PCI and moderate to severe non-IRA stenosis (SS ≥2.5) have more subsequent cardiac events. Those populations should be treated with more aggressive preventive and medical management.

## Introduction

Acute thrombotic occlusion of a coronary artery is the leading cause of ST-segment elevation myocardial infarction (STEMI) [1]. Primary percutaneous coronary intervention (PCI) is currently the preferred therapy for restoring perfusion of the infarct-related artery (IRA), also known as the culprit artery[2], [3]. Between 40 and 65% of patients treated with primary PCI for STEMI have multi-vessel disease (MVD)[4]–[6], which is an independent predictor of long-term mortality in these patients[7], [8]. Studies have indicated that MVD with chronic total occlusion (CTO) is a risk factor associated with a worse outcome in STEMI patients who undergo primary PCI. However, the association between the severity of non-IRA lesions and mortality in STEMI patients has not been elucidated.

The SYNTAX score (SS) is an angiographic scoring tool for systematically quantifying the severity and assessing the characteristics of each coronary lesion[9]. It is used worldwide to predict long-term outcomes in patients with coronary artery disease undergoing elective PCI or coronary artery bypass graft surgery[10], [11]. The SS is also useful for predicting short- and long-term outcomes in patients with STEMI who are treated with primary PCI[12]–[15].

The aim of our study was to quantify and assess the severity of non-IRA lesions calculated by SS, and to determine the impact of the severity of non-IRA in patients presenting with STEMI and treated with primary PCI.

## Materials and Methods

### Subjects

This study was conducted in accordance with the Declaration of Helsinki and was approved by the Institutional Review Board of Mackay Memorial Hospital. The patient records and information were anonymized and de-identified prior to analysis. Three hundred twenty-three consecutive patients undergoing primary PCI for STEMI at a single tertiary center between August 2009 and December 2012 were included in this analysis. Acute STEMI was defined as typical chest pain lasting for>30 min within the last 12 h, with electrocardiographic findings of ST elevation>1 mm in at least two consecutive leads or new-onset left bundle branch block, and elevation of serum levels of troponin-I or the MB fraction of creatine kinase. The diagnosis was confirmed by coronary angiography in all patients. We excluded from the study patients who reported a previous MI within 6 months, previous coronary artery bypass surgery, symptom onset more than 12 h before, pretreatment with thrombolytic therapy before primary PCI, previous hemodialysis, sepsis, neoplasm, hematological disorders, or acute stroke during the course of their hospital stay. Treatment of complications such as ventricular arrhythmia, cardiogenic shock, and cardiac arrest was administered according to guidelines.

### Data Collection and Definitions

Demographic data, disease history (such as hypertension [HTN] or diabetes mellitus [DM]), current tobacco use, coronary angiographic results, and prescribed medications were obtained from the hospital medical registry. The blood total cholesterol, high-density lipoprotein cholesterol, low-density lipoprotein cholesterol (LDL-C), triglyceride (TG), and glycated hemoglobin (HbA1c), and creatinine levels were evaluated on the same day that the patients underwent primary PCI. All blood samples were collected by venipuncture after at least 8 h of fasting. HTN was defined as a history of HTN, a systolic BP of ≥140 mmHg, or a diastolic BP of ≥90 mmHg. Patients were defined as having DM if they had a history of DM, HbA1c levels ≥6.5%, or if they were using oral hypoglycemic agents or insulin. All patients received 300 mg aspirin and 300 mg clopidogrel orally, and were given a bolus of intravenous unfractionated heparin (75–100 U/kg) prior to primary PCI. At the discretion of the attending interventional cardiologist, glycoprotein IIb/IIIa inhibitors were administered as adjunctive therapy. Coronary angiography was performed using a Philip Integris BH 5000 device equipped with the cardiovascular (CV) angiography analysis system CAAS II (Best, Netherlands). The SS was calculated retrospectively by two trained operators who were blinded to the patients' demographics and outcomes. The SS was determined for all coronary lesions with>50% diameter stenosis in a vessel>1.5 mm, based on the SYNTAX Score Calculator 2.1 (www.syntaxscore.com). The pervious studies demonstrated both IRA SS calculated before any intervention preformed and IRA SS calculated after flow restoration were independent predictors of clinical outcomes[13], [14], but how to evaluate SS after intervention is inconclusive. Therefore, total SS, SS of the IRA, and SS of non-IRA were all calculated before any intervention preformed in our study. The non-IRA SS was calculated as the sum of the SS in all non-culprit coronary arteries of MVD.

### Clinical outcomes

The outcome measure in the current analysis was the time from the date of primary PCI until the first occurrence of a component of the composite endpoint: all-cause death, CV death (caused by MI, refractory heart failure, or ventricular arrhythmia), reinfarction (fatal or non-fatal MI), target lesion revascularization for myocardial ischemia, or stroke. Major adverse CV events (MACE) were defined as the composite of CV death, reinfarction (fatal or non-fatal MI), target vessel revascularization for ischemia, or stroke. Follow-up for all patients was continued until December 31, 2013.

### Statistical Analysis

Results are expressed as mean ± SD or as percentages. Student's *t* test was used to compare differences between groups for continuous variables, and the chi-square test was employed for categorical data. Receiver operating characteristic (ROC) curve analysis is the most common technique used for assessing diagnostic tests and to identify a cutoff point[16]. In this study, we want to chose the cutoffs by MACE as the outcome measure to discriminate the value of IRA and non-IRA Syntax Score proposed to be used as decisional levels in clinical practice when it is necessary to revascularize the non IRA. According to the ROC curve, we were able to define the cutoff point for the SS for IRA and non-IRA to maximize the clinical sensitivity and specificity of the test. We used the cutoff point as a criterion for the classification of the severity of non-IRA lesions. A Cox proportional hazards model was used to calculate hazard ratios (HRs) to determine the factors contributing to all-cause death, CV death, and MACE. The HRs (95% confidence intervals [CIs]) were adjusted for sex, age, HTN, DM, smoking status, LDL-C level (<100 mg/dL versus ≥100 mg/dL), IRA SS (<10.25 versus ≥10.25), and non-IRA SS (<2.5 versus ≥2.5). Kaplan–Meier survival curves were constructed and compared using the log-rank test. A P-value <0.05 was considered significant. All statistical analyses were performed using SPSS software, version 19 (IBM SPSS Statistics, State of New York) and STATA (version 11.0, College Station, Texas).

## Results

### Patient characteristics

**Figure 1:**
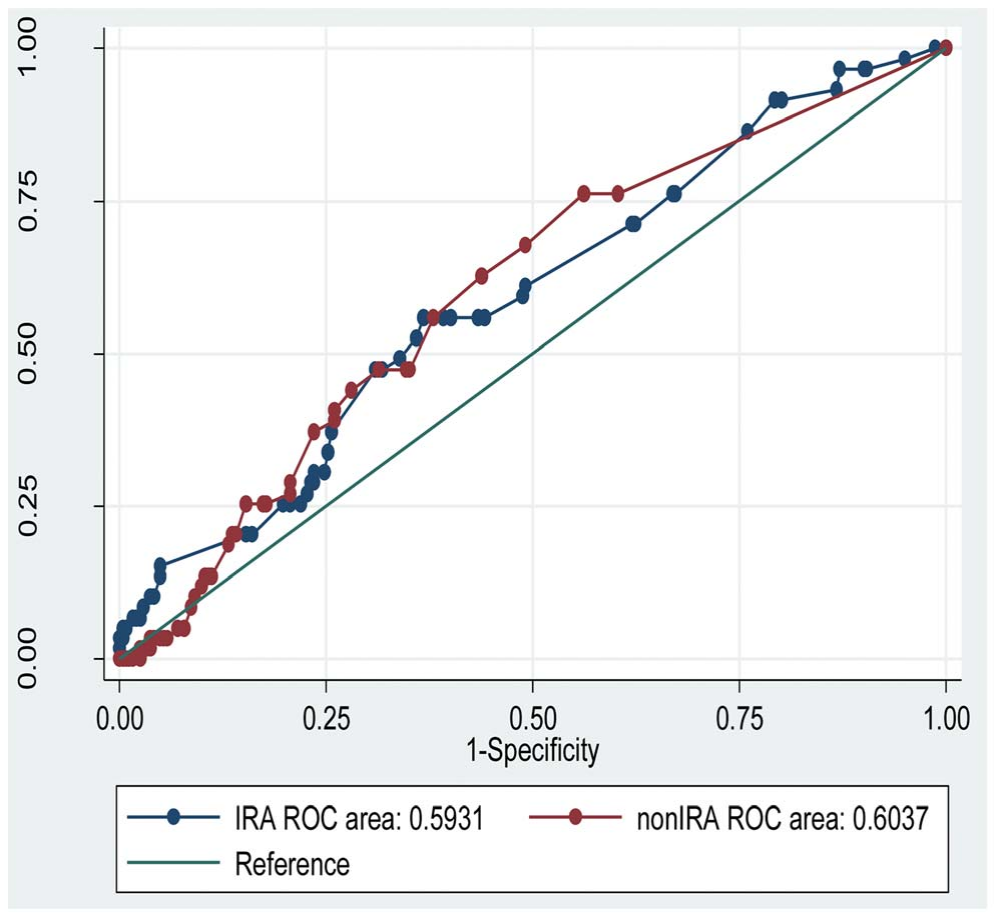
Receiver operating characteristic (ROC) curve analysis and cutoff value for the severity of stenosis of infarcted and non-infarcted related arteries in patients with acute ST-elevation myocardial infarction (STEMI).

A total of 323 patients were initially considered for study inclusion. Ten were excluded because no complete diagnostic coronary angiogram was available and another 2 because they had previously undergone coronary bypass grafting. Survival status and follow-up could not be obtained in 10 foreign patients. Overall, a total of 301 consecutive patients were included in our study for analysis. A Mean of SS in IRA and non –IRA was 12. 8 ± 0.4 and 6.2 ± 0.5, respectively. A median of SS in IRA and non –IRA was 11 and 3, respectively. Firstly, we used ROC to determine the appropriate cutoff value for severity of non-IRA lesions that corresponded to MACE (Figure 1). The closer the ROC curve to the upper-left corner, the higher the predictive power for predicting MACE. The optimal cutoff point for non-IRA SS was 2.5, with a sensitivity and specificity for MACE of 68% and 51%, respectively. The optimal cutoff point of IRA SS was 10.25, with a sensitivity and specificity for MACE of 61% and 50%, respectively. The area under the ROC curve (AUC) did not differ between IRA and non-IRA lesions (P  =  0.85).

Since we were investigating the association between the severity of non-IRA lesions and clinical outcomes, we divided the patients at the cutoff point for non-IRA SS of 2.5, yielding subgroups with no/mild non-IRA stenosis (SS <2.5) and moderate/severe non-IRA stenosis (SS ≥2.5). Table 1 shows the baseline characteristics of these subgroups. Patients who had moderate/severe non-IRA stenosis were more likely to have HTN as comorbidity (66.5% vs. 47.6%, P  =  0.001). Patients with moderate/severe non-IRA stenosis had a higher total SS than those with no/mild non-IRA stenosis (23.8 ± 10.3 vs. 13.7 ± 7, P <0.001). There were no differences in intra-aortic balloon pumping support, use of temporary pacemaker or extracorporeal membrane oxygenation, prescribed medication, Killip classification, IRA SS, or IRA location between patients with no/mild non-IRA stenosis and those with moderate/severe non-IRA stenosis. Compared to those with moderate/severe non-IRA stenosis, patients with no/mild non-IRA stenosis had a lower incidence of MACE (13.3% vs. 25.3%, P  =  0.009), CV mortality (2.8% vs. 8.3%, P  =  0.04), and all-cause mortality (3.5% vs. 10.1%, P  =  0.02).

**Table 1:** Clinical and angiographic characteristics of patients with no- mild non- IRA stenosis and moderate-severe non- IRA stenosis.

	no- mild non- IRA stenosis (N = 143)	moderate-severe non- IRA stenosis (N = 158)	P value
	**SYNTAX score<2.5**	**SYNTAX score≥ 2.5**	
Age (years)	58.8 ± 13.6	60.3 ± 12.9	0.32
Gender (men,%)	120 (83.9%)	137(86.7%)	0.46
Smoking status (yes,%)	89 (62.2%)	98 (62.0%)	0.97
HTN(yes,%)	68 (47.6%)	105 (66.5%)	0.001
DM(yes,%)	42 (29.4%)	63 (39.9%)	0.06
VT(yes,%)*	14 (9.8%)	12 (7.6%)	0.50
CPCR(yes,%)*	11 (7.7%)	14 (8.9%)	0.71
TC (mg/dL)	183.3 ± 44.1	176.7 ± 49.2	0.23
TG (mg/dL)	167.8± 153.1	167.8 ± 191.3	1.00
LDL-C (mg/dL)	112.7 ± 32.5	112.7 ± 35.1	1.00
**Device use**			
IABP(yes,%)	18 (12.6%)	27 (17.1%)	0.27
TPM(yes,%)	8 (5.6%)	14 (8.9%)	0.28
ECMO(yes,%)	1 (0.7%)	3 (1.9%)	0.35
**Medication**			
GpIIbIIIa	70 (49.3%)	66 (41.5%)	0.18
Enoxaprine	138 (97.2%)	152 (96.2%)	0.46
Aspirin	138 (97.2%)	151 (95.6%)	0.33
Clopidogrel	139 (97.9%)	148 (93.7%)	0.05
ACEI/ARB	129 (90.8%)	135 (85.4%)	0.12
Beta-Blocker	107 (75.4%)	113 (71.6%)	0.40
Statin	128 (90.1%)	133 (84.2%)	0.10
**Killip classification**			0.46
Killip I	77 (53.8%)	71 (44.9%)	
Killip II	31 (21.7%)	38 (24.1%)	
Killip III	7 (4.9%)	11 (7.0%)	
Killip IV	28 (19.6%)	38 (24.1%)	
**IRA location**			0.29
Left main	2 (1.4%)	2 (1.3%)	
LAD	80(55.9%)	73(46.2%)	
LCX	7(4.9%)	14 (8.9%)	
RCA	54 (37.8%)	69 (43.7%)	
Total SS	13.7 ± 7	23.8 ± 10.3	<0.001
IRA SS	13.2 ± 7.0	12.4 ± 6.7	0.31
Non IRA SS	0.5 ± 1.9	11.4 ± 8.5	<0.001
**Clinical outcomes**			
All cause mortality	5 (3.5%)	16 (10.1%)	0.02
CV death	4 (2.8%)	13 (8.3%)	0.04
MACE	19 (13.3%)	40 (25.3%)	0.009
TLR	10 (7.0%)	19 (12.0%)	0.14
Re-infarction	6 (4.2 %)	17 (10.8%)	0.03
stroke	3 (2.1%)	4 (2.5%)	1.00

Abbreviation: HTN: hypertension; DM: diabetes mellitus; VT: ventricular tachycardia; CPCR: cardiopulmonary cerebral resuscitation;TC: total cholesterol; TG: triglyceride; LDL-C: low density lipoprotein cholesterol; IABP: intra aortic balloon pumping; TPM: temporary pacemaker; ECMO: extracorporeal membrane oxygenation; Gp: Glyoproein; ACEI: angiotensin converting enzyme; ARB: angiotensin receptor blocker; IRA: infarcted related artery; LAD: left descending artery; LCX: left circumflex; RCA: right coronary artery; SS: SYNTAX score; CV: cardiovascular; MACE: major adverse cardiovascular events; TLR: target lesion revascularization.

*: occurred before primary percutaneous coronary intervention.

### Clinical outcomes

All patients received clinical follow-up with a median duration of 580 days. A total of 80 endpoints occurred during follow-up: 59 (19.6%) new CV events and 21 (7.0%) deaths. Patients who had non-IRA CTO had a higher MACE rate of 8.0 % (versus 6.9 % for those who had no non-IRA CTO; p  =  0.834); a higher CV mortality of 8.0% (versus 5.5 % for those who had no non-IRA CTO; p =  0.609); a higher MACE of 24 % (versus 19.2 % for those who had no non-IRA CTO; p  =  0.563). The rate of all cause mortality in patients with triple vessel disease (TVD), double vessel disease (DVD) and single vessel disease (SVD) was 12.6%, 5.8%, and 3.6% (p  =  0.04). The rate of MACE in patients with TVD, DVD and SVD was10.6%, 4.9% and 2.8% (p  =  0.01). The rate of CV mortality patients with TVD, DVD and SVD was 10.6%, 4.9% and 2.8% (p  =  0.06). The Cox proportional hazards regression model was used for multivariate analysis of MACE, all-cause mortality, and CV mortality after acute STEMI. The independent variables of the regression model included age, sex (men vs. women), current smoking status, HTN, DM, LDL-C (≥100 vs. <100 mg/dL), IRA SS (≥10.25 vs. <10.25) and non-IRA SS (≥2.5 vs. <2.5). The predictors of MACE, all-cause mortality, and CV mortality are shown in Table 2. After adjustment for the parameters mentioned above, non-IRA SS of ≥2.5 vs. <2.5 (adjusted HR [AHR]: 2.15, 95% CI: 1.21–3.79, P  =  0.008) was an independent predictor of MACE. DM (AHR: 3.04, 95% CI: 1.03–8.99, P  =  0.04), an LDL-C level of ≥100 vs. <100 mg/dL (AHR: 0.29, 95% CI: 0.10–0.84, P  =  0.02), and a non-IRA SS of ≥2.5 vs. <2.5 (AHR: 3.49, 95% CI: 1.13–10.8, P  =  0.03) were independent predictors of all-cause mortality. DM (AHR: 7.64, 95% CI: 1.63–35.8, P  =  0.01) was an independent predictor of CV mortality, but the non-IRA SS of ≥2.5 vs. <2.5 (AHR: 3.59, 95% CI: 0.90–12.08, P  =  0.07) only showed a trend to predict CV mortality, with borderline statistical significance.

**Table 2:** Cox regression analysis for major adverse cardiovascular events, all cause mortality and cardiovascular mortality.

	MACE		All cause mortality		CV mortality	
	hazard ratio (95% CI)	P value	hazard ratio (95% CI)	P value	hazard ratio (95% CI)	P value
Age (years)	1.02 (1.00–1.05)	0.07	1.04 (0.99–1.08)	0.10	1.02 (0.97–1.07)	0.49
gender(men vs women)	0.82(0.35–1.92)	0.65	1.36 (0.39–4.72)	1.00	10.2(0.24– 4.37)	0.98
HTN (yes vs no)	0.76 (0.43–1.34)	0.34	1.29 (0.39–4.18)	0.68	1.26 (0.33–4.82)	0.74
DM (yes vs no)	1.37 (0.78–2.43)	0.28	3.04 (1.03–8.99)	0.04	7.64(1.63–35.8)	0.01
Smoking status (yes vs no)	0.93 (0.50–1.74)	0.83	1.13 (0.36–3.06)	0.83	0.93 (0.27–3.23)	0.90
LDL (≥100 vs <100 mg/dL)	0.61 (0.35–1.05)	0.07	0.29 (0.10–0.84)	0.02	0.44 (0.15–1.35)	0.15
**Non-IRA SS (≥2.5 vs <2.5)**	2.15(1.21–3.79)	**0.008**	3.49 (1.13–10.8)	**0.03**	3.29 (0.90–12.08)	0.07
IRA SS (≥10.25 vs <10.25)	1.6 (0.93–2.85)	0.08	1.46(0.56–3.83)	0.44	1.57(0.51–4.80)	0.43

Abbreviation as table 1; CI: confidence interval.

**Figure 2:**
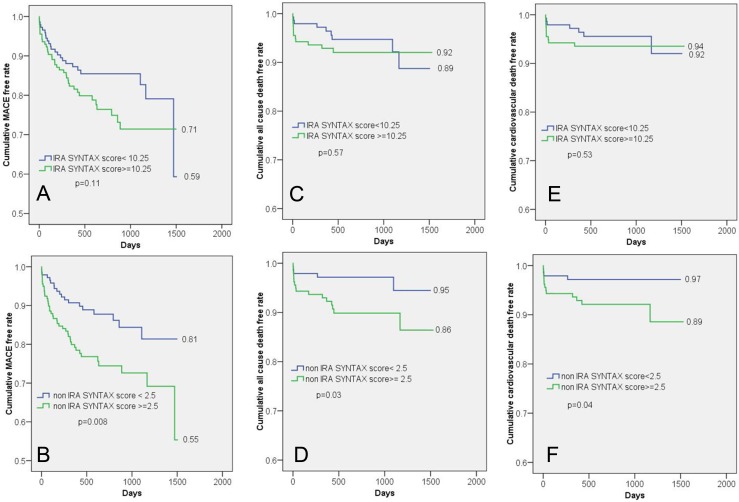
Kaplan–Meier analysis of major adverse cardiovascular events (MACE), all-cause mortality, and cardiovascular mortality in all patients, subdivided according to cutoff levels for non-IRA SS (2.5) and IRA SS (10.25). (A) Cumulative MACE-free rate between patients with IRA SS ≥10.25 and <10.25 (B) Statistical significance of the difference in cumulative MACE-free rate between patients with IRA SS ≥2.5 and <2.5 (C) Cumulative all-cause mortality-free rate between patients with IRA SS ≥10.25 and <10.25 (D) Statistical significance of the difference in cumulative all-cause mortality-free rate between patients with IRA SS ≥2.5 and <2.5 (E) Cumulative cardiovascular mortality-free rate between patients with IRA SS ≥10.25 and <10.25 (F) Statistical significance of the difference in cumulative cardiovascular mortality-free rate between patients with IRA SS ≥2.5 and <2.5.

Since we found that non-IRA SS was a shared and strong predictor of MACE, Kaplan–Meier analysis was performed to examine the univariate association between the two subgroups of non-IRA SS scores (≥2.5 vs. <2.5), IRA SS scores (≥10.25 vs. <10.25), and the outcomes of the cohort (Figure 2). The patients with no/mild non-IRA stenosis (non-IRA SS <2.5) exhibited a significantly lower rate of MACE, all-cause mortality, and CV mortality than those with moderate/severe non-IRA stenosis (SS ≥2.5; 81%, 95%, 97% vs. 55%, 86%, 89%, respectively; P <0.05). However, there was no difference in MACE, all-cause mortality, or CV mortality between patients with IRA SS ≥10.25 and those with IRA SS <10.25.

## Discussion

Our study demonstrates that the extent of non-IRA stenosis is an independent predictor of long-term all-cause mortality and MACE after adjustment for confounding variables. Our overall primary PCI mortality is in agreement with published data[17]. In our study, 36.5% of patients with MI had non-IRA stenosis: a result similar to those of previous studies[4]–[8], [15], [18]. Prior studies in the primary PCI era indicated that MVD was a significant predictor of poor outcomes in patients undergoing primary PCI, compared with single-vessel disease (SVD)[19], [20]. Sorajja et al demonstrated that MVD was associated with a higher rate of IRA and non-IRA revascularization in patients with STEMI after primary PCI^18^. Furthermore, the presence of CTO in a non-IRA is associated with worse outcomes in patients undergoing primary PCI for acute STEMI[8], [21]-[23]. In those studies, MVD was an independent factor for CV events, and the severity of non-IRA lesions seemed to play a role in contributing to CV events. In a post hoc analysis of the Harmonizing Outcomes with RevascularIZatiON and Stents in Acute Myocardial Infarction trial, the CV event rate was found to be higher in patients with MVD and non-IRA CTO than in those with SVD[8]. Our study found similar results. However, the contribution of the severity of IRA and non-IRA stenosis to the outcome of STEMI patients treated with primary PCI was not elucidated. Therefore, we considered the IRA and non-IRA SS independently, to investigate which component had an impact on the prognosis. Our analysis demonstrated that patients with moderate/severe non-IRA stenosis (score ≥2.5) had a higher incidence of MACE, CV mortality, and all-cause mortality than those with no/mild non-IRA stenosis (score <2.5), but the same result was not found for IRA SS. Our study indicated that patients with moderate/severe non-IRA stenosis might need more aggressive treatment.

The mechanisms underlying the greater frequency of CV events in patients with moderate/severe non-IRA stenosis are multifactorial. First, in our study the patients with moderate/severe non-IRA stenosis had a higher prevalence of comorbidities (such as HTN and DM) compared to those with no/mild non-IRA stenosis. Second, MVD was a significant predictor of a poor outcome after primary PCI, compared with SVD[8], [18], [19]. Our study showed that there was almost a twofold increase in the relative risk of MACE for those with moderate/severe non-IRA stenosis compared to those with no/mild non-IRA stenosis. In one study, nearly 10% of STEMI patients needed subsequent PCI in the non-IRA during a follow-up of up to 3 years[24]. Third, patients presenting with a higher SS might be exposed to complicated primary PCI procedures, including treatment of bifurcations or left main disease, with a resulting effect on the clinical outcome, and might require repeated revascularization because of ischemia caused by restenotic lesions in areas of high intervention complexity. The explanation for the high late mortality in patients with moderate/severe non-IRA stenosis could be that they are potentially at a higher risk from the initial acute STEMI. The area at risk from the ischemia would be more extensive in patients with moderate/severe non-IRA stenosis than in those with no/mild non-IRA stenosis. Our findings highlight the fact that non-IRA disease at presentation may not be benign.

The finding that the severity of non-IRA stenosis adds an incremental risk of adverse outcomes in patients with STEMI undergoing primary PCI may have important clinical implications. Based on previous studies, PCI in non-infarct lesions does not show a benefit in terms of reducing death and MI[25], [26]. Current guidelines indicate simultaneous treatment of multiple vessels during acute STEMI be performed only in cases of cardiogenic shock[3], whereas staged PCI procedures demonstrate better outcomes in STEMI patients than in those with multiple vessel PCI[27], [28]. In contrast, the Preventive Angioplasty in Acute Myocardial Infarction study reported that the primary outcome of cardiac death, MI, or refractory angina was significantly less common in the preventive-PCI group, as compared with optimal medical therapy alone[29]. Therefore, the best strategy for staged revascularization in STEMI with MVD to improve long-term prognosis still needs to be clarified by further clinical trials.

Several limitations of the current study should be mentioned. The main one is that our study was a retrospective observational study from a single center and not a randomized prospective study. The true incidence of CV events could not be estimated from our study. The study used MACE as the outcome measure to discriminate the value of IRA and non-IRA Syntax Score. Since the cutoff is derived by the data themselves, this almost certainly leads to over-estimates of performances such as sensitivity, specificity, positive and negative predictive values. Additionally, our study did not evaluate subsequent revascularization attempts in the non-IRA. Because of the limited sample size and its being a single hospital study, the patients in our study might not be representative of the entire population of acute STEMI patients who undergo primary PCI. In order to confirm our findings, a study with a larger sample of patients is required. However, the results of our analysis should be considered hypothesis generating.

In conclusion, our finding indicate that STEMI patients who are treated with primary PCI and have moderate/severe non-IRA stenosis (score ≥2.5) suffer more subsequent MACE, suggesting that those populations should be treated with more aggressive preventive and medical management.
